# Non-native molluscan colonizers on deliberately placed shipwrecks in the Florida Keys, with description of a new species of potentially invasive worm-snail (Gastropoda: Vermetidae)

**DOI:** 10.7717/peerj.3158

**Published:** 2017-04-05

**Authors:** Rüdiger Bieler, Camila Granados-Cifuentes, Timothy A. Rawlings, Petra Sierwald, Timothy M. Collins

**Affiliations:** 1 Integrative Research Center, Field Museum of Natural History, Chicago, IL, United States; 2 Mote’s Tropical Research Laboratory, Summerland Key, FL, United States; 3 Department of Biological Sciences, Florida International University, Miami, FL, United States; 4 Department of Biology, Cape Breton University, Sydney, Nova Scotia, Canada

**Keywords:** Colonization, Diversity, Western Atlantic, Invasive species, Coral reef, Artificial reef, Introductions, Fouling community

## Abstract

Artificial reefs created by deliberately sinking ships off the coast of the Florida Keys island chain are providing new habitat for marine invertebrates. This newly developing fouling community includes the previously reported invasive orange tube coral *Tubastraea coccinea* and the non-native giant foam oyster *Hyotissa hyotis*. New SCUBA-based surveys involving five shipwrecks spanning the upper, middle, and lower Florida Keys, show *T. coccinea* now also established in the lower Keys and *H. hyotis* likewise extending to new sites. Two additional mollusks found on the artificial reefs, the amathinid gastropod *Cyclothyca pacei* and gryphaeid oyster *Hyotissa mcgintyi*, the latter also common in the natural reef areas, are discussed as potentially non-native. A new species of sessile, suspension-feeding, worm-snail, *Thylacodes vandyensi*s Bieler, Rawlings & Collins n. sp. (Vermetidae), is described from the wreck of the *USNS Vandenberg* off Key West and discussed as potentially invasive. This new species is compared morphologically and by DNA barcode markers to other known members of the genus, and may be a recent arrival from the Pacific Ocean. *Thylacodes vandyensis* is polychromatic, with individuals varying in both overall head-foot coloration and mantle margin color pattern. Females brood stalked egg capsules attached to their shell within the confines of their mantle cavity, and give rise to crawl-away juveniles. Such direct-developing species have the demonstrated capacity for colonizing habitats isolated far from their native ranges and establishing rapidly growing founder populations. Vermetid gastropods are common components of the marine fouling community in warm temperate and tropical waters and, as such, have been tagged as potentially invasive or with a high potential to be invasive in the Pacific Ocean. As vermetids can influence coral growth/composition in the Pacific and have been reported serving as intermediate hosts for blood flukes of loggerhead turtles, such new arrivals in the Florida Keys National Marine Sanctuary are of concern. Growing evidence indicates that artificial reefs can act as permanent way-stations for arriving non-natives, providing nurseries within which populations may grow in an environment with reduced competition compared to native habitats. Consequently, artificial reefs can act as sentinels for the appearance of new species. Ongoing monitoring of the developing molluscan fauna on the artificial reefs of the Florida Keys is necessary to recognize new invasions and identify potential eradication targets, thereby assuring the health of the nearby natural barrier reef.

## Introduction

Artificial reefs are anthropogenic structures that are deliberately or accidentally submerged, and often alter local habitat by providing vertical relief and a hard substratum where typically none previously existed (Bohnsack & Sutherland, 1985; Sheehy & Vik, 2010). In the Florida Keys, an island chain at the southernmost tip of Florida that is home to the world’s third-largest barrier reef system, reef environments are exposed to a wide range of environmental and anthropogenic stressors (Donahue et al., 2008). Consequently, the development of artificial reefs is viewed as one avenue to help mitigate human impacts on the natural reef environment (e.g., Harris & Woodring, 2001). The regional artificial marine substrata range from concrete and metal surfaces (as part of bridges, causeways, seawalls, lighthouses, and other navigational markers), to legally deployed wooden/plastic/concrete crayfish and stone crab traps (and their rogue versions after storm displacement), illegally placed artificial habitats to attract spiny lobsters (“casitas”), and shipwrecks, the latter both accidentally and intentionally sunk.

**Figure 1:**
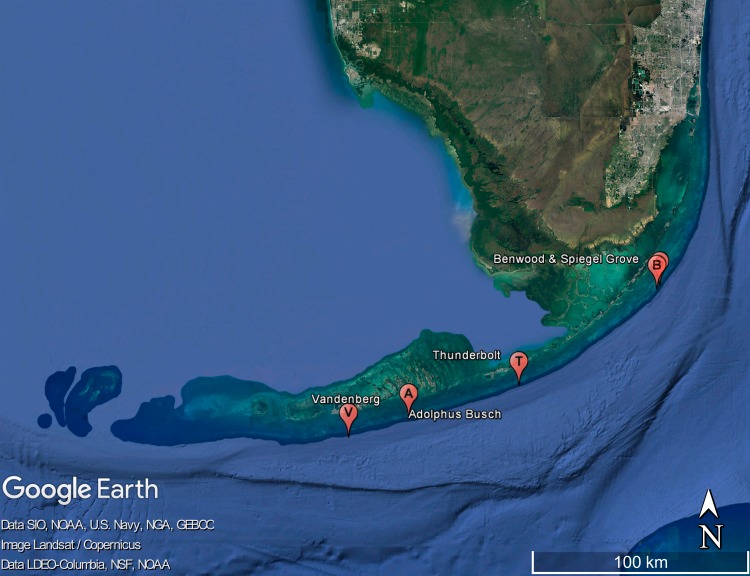
Map of the Florida Keys showing the locations of the five surveyed shipwrecks just south of the outer barrier reef line. City of Miami in the upper right, Dry Tortugas archipelago in the lower left. Map data ©2016 Google, Image Landsat/Copernicus, Data SIO, NOAA, US Navy, NGA, NSF, GEBCO, LDEO-Columbia.

Florida has one of the most active artificial reef programs in the United States (Dodrill & Fletcher, 2012), with at least half of the nation’s human-made marine reefs within its border and a focus on developing recreational fisheries and the enhancement of SCUBA diving activities (Seaman, 2004). In the Florida Keys, artificial reefs primarily consist of shipwrecks. In addition to numerous vessels that foundered off the Florida Keys during storms or for other reasons over the centuries and remain as accidental wrecks, other vessels were deliberately placed on the ocean bottom. The latter include many ships used during missile tests and naval exercises, especially from the 1940s to the 1970s, with the wrecks of this so-called ‘Key West Ghost Fleet’ mostly lying in fairly deep water (56–128 m) between the island of Key West and the Dry Tortugas archipelago. Beginning in the 1960s, large vessels including decommissioned United States Coast Guard cutters were sunk in what is now the Florida Keys National Marine Sanctuary (FKNMS), to serve as artificial reefs. Four of these are formal parts of the ‘Florida Keys Shipwreck Trail’ (National Ocean Service, 2015). After the establishment of the FKNMS in the 1990s, several other ships were scuttled in the region, including the *USS Spiegel Grove* (2002, off Key Largo, upper Florida Keys), the *Adolphus Busch* (1998, off Summerland Key, middle Florida Keys), and the *USNS General Hoyt S. Vandenberg* (2009, off Key West, lower Florida Keys); see Fig. 1. These are very large vessels, with the 155-meter long *Spiegel Grove* holding the record as the largest artificial reef at the time of her sinking and the 160-meter long *Vandenberg* currently representing the second-largest artificial reef in the world (the largest now being the aircraft carrier *USS Oriskany*, placed in the Gulf of Mexico south off Pensacola, Florida). To provide sport diving attractions in accessible depth, these deliberately placed vessels usually rest about 10 km offshore, on sand bottoms in 34–44 m depth, with their superstructures extending into shallower water. Artificial reefs in the northern Gulf of Mexico, which are formed by numerous oil and gas platforms (“rigs to reefs,” e.g., Sammarco et al., 2014), are positioned in waters that until their introduction was nearly devoid of shallow hard substrata (Atchison, 2005) except for those in the vicinity of the Flower Garden Banks reefs (Sammarco, Atchison & Boland, 2004). In contrast, the string of shipwrecks off the Florida Keys is in close proximity to the living barrier reef.

The potential impacts of such artificial reef placements in what is recognized as a high priority region for biodiversity protection (Jenkins & Van Houtan, 2016) are topics of interest and concern. Artificial reefs in the Florida Keys are known to have substantive positive impacts of the local economy by benefitting dive charter and associated industries (Leeworthy, 2011; Johns, 2012). They can also reduce user pressure on the adjacent natural reefs as reported for one of the regional wrecks, the *Spiegel Grove* (Leeworthy, Maher & Stone, 2006), although this was not true for the *Vandenberg*, where the total use on both natural and artificial reefs increased after placement of the wreck (Leeworthy, 2011). Artificial reefs can effectively accumulate fish and other organisms (e.g., Bohnsack & Sutherland, 1985; Wilson et al., 2001), and may create corridors for further dispersal and expansion of both native and non-native species (Atchison, 2005; Sheehy & Vik, 2010; Broughton, 2012; Precht et al., 2014). The attraction of non-native (and potentially invasive) species is chief among “the unintended consequences of artificial reef construction and siting” (Lindberg & Seaman, 2011: 54). Formal monitoring of artificial reefs in Florida varies locally and concentrates on physical (e.g., temperature, reef movement), socio-economic (e.g., expenditures, usage), or biological impacts (e.g., species abundance) (e.g., Seaman, 2004). On the wrecks of the Florida Keys, formal biological monitoring has concentrated on fish populations (e.g., Akins & Semmens, 2011), and most discussions of aquatic invasive species in the Florida Keys have focused on the lion fish species, *Pterois volitans* (Linnaeus, 1758) and *P. miles* (Bennett, 1828) (e.g., Donahue et al., 2008; Schofield, 2009). Data on non-native invertebrates on the artificial reefs have remained anecdotal and, not surprisingly, are limited to a few species that stand out from the native fauna by their color or size.

**Figure 2:**
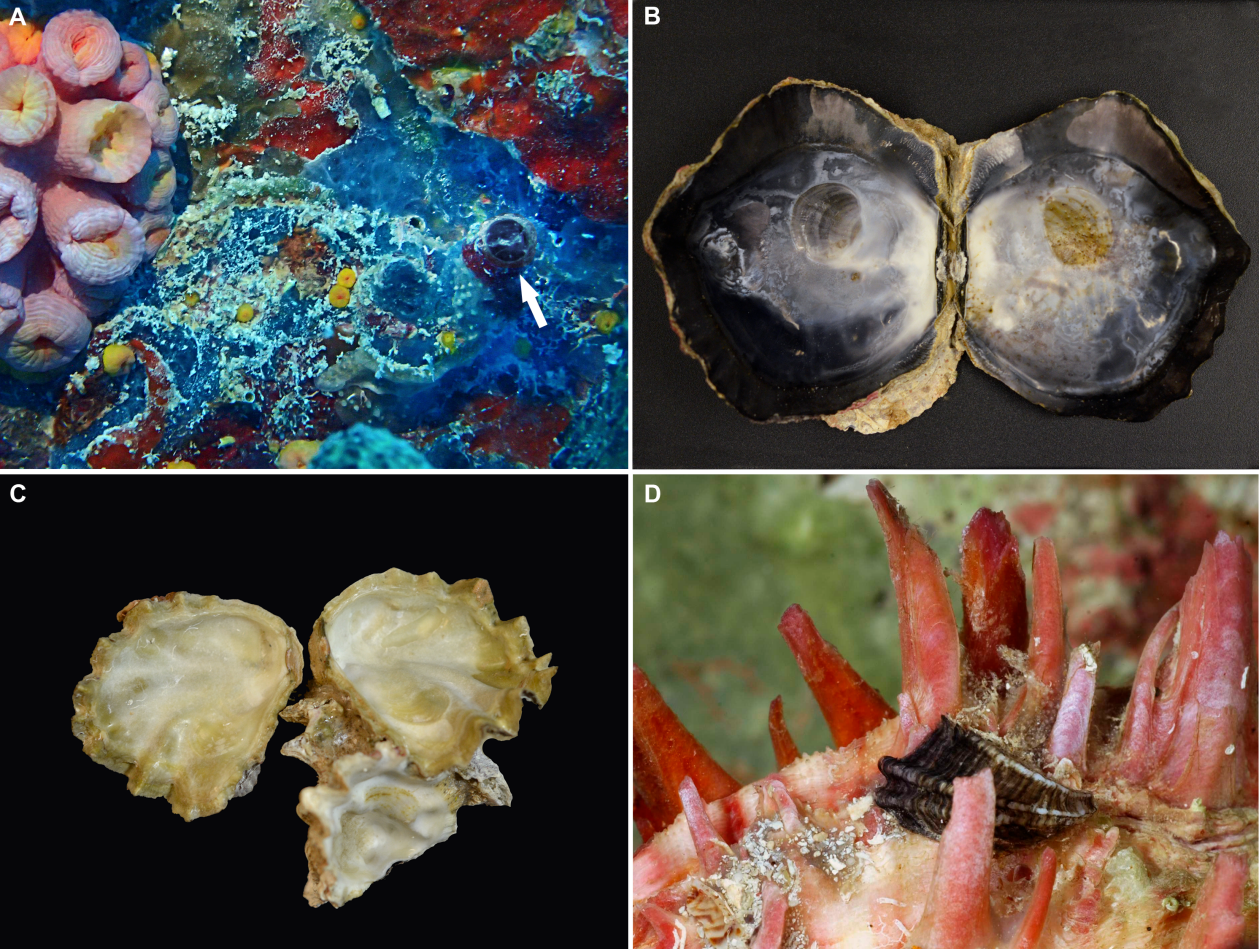
Non-native and potentially non-native invertebrate species observed and collected on the wrecks off the Florida Keys. (A) Orange tube coral *Tubastraea coccinea* (left) and solitary adult specimen of the vermetid gastropod *Thylacodes vandyensis* n. sp. (arrow); *Vandenberg* wreck, photographed with artificial light at about 29 m depth on the side of the ship’s superstructure, September 2016; diameter of vermetid tube opening approximately 5.0 mm. (B) Interior shell view of gryphaeid oyster *Hyotissa hyotis*; first record from the *Spiegel Grove* wreck, from hull in 31 m (FMNH 335064; August 2012; greatest valve diameter 150.0 mm). (C) Small cluster of gryphaeid oyster *Hyotissa mcgintyi*; the most abundant bivalve on the *Vandenberg* wreck in about 29 m depth, serving as substratum for many other species of the fouling community (FMNH 344618; September 2016; greatest valve diameter of opened specimen 50.0 mm). (D) Specimen of the cap-shaped amathinid gastropod *Cyclothyca pacei* near the rim of the lower valve of an Atlantic thorny oyster, *Spondylus americanus*, from the *Thunderbolt* wreck, from 29–37 m depth, August 2003; photographed alive (note black periostracum on the white shell) after brushing off red encrusting sponge; length of gastropod shell, 11.5 mm (FMNH 301979).

The most visible among these is the orange cup coral, *Tubastraea coccinea* Lesson, 1829, an Indo-Pacific scleractinian species that has spread widely in the Atlantic (Cairns, 2000; Creed et al., 2016) and was first reported from Florida by Fenner & Banks (2004), where artificial structures appear to be its preferred habitat. The authors cited records for this species from artificial reef sites in the Florida Keys, including the *USCGC William J. Duane* (off Key Largo, upper Florida Keys). Since then, large populations have been reported on the wrecks of the *Duane* and the nearby *Spiegel Grove* (Shearer, 2010). A study by Ferry (2009) indicated that the species had not yet become established in the lower Florida Keys, however according to our own observations (January 2016; Fig. 2A), *Tubastraea coccinea* is now well established on the *Vandenberg* wreck off Key West.

Herein we review the occurrence of non-native, and potentially non-native, fouling invertebrates on artificial (shipwreck) reefs in the Florida Keys and document the regional occurrences of the foam oysters *Hyotissa hyotis* and *H. mcgintyi*, as well as the gastropod *Cyclothyca pacei.* We also describe a new vermetid gastropod species, *Thylacodes vandyensis* n. sp., recently discovered on these artificial substrata.

## Materials and Methods

Molluscan fauna of natural reefs along the Florida Keys have been sampled by SCUBA intermittently over the past 30 years. Artificial reefs, the focus of this study, have been investigated since 2002 (as individually dated below), with specimens of the newly described vermetid gastropod species taken in May of 2014 and September of 2016. Artificial reef data presented herein are based on brief SCUBA-assisted surveys conducted by RB and PS on the following wrecks, arranged from NE to SW (see Fig. 1): the *USS Spiegel Grove* (a former US Navy dock landing ship intentionally sunk in 2002 off Key Largo, upper Florida Keys; 25°04′02′′N, 80°19′08′′W; sampled as FK-1026 in August 2012); the *SS Benwood* (a Norwegian merchant freighter that sunk after collision in 1942 and broke up subsequently; 25°03.144′N, 080°19.93′W; sampled as FK-1027 in August 2012); the *Thunderbolt* (originally a US Army mine planting ship and later modified as a lightning strike research vessel, intentionally sunk in 1986 off Key Vaca, middle Florida Keys; 24°39.663′N, 080°57.784′W; sampled as FK-717 in August 2003); the *Adolphus Busch* (a former cargo ship intentionally sunk in 1998 off Ramrod Key, lower Florida Keys; 24°31.841′N, 081°27.688′W; sampled as FK-908 in August 2009 and as FK-991 in November 2011); and the *USNS General Hoyt S. Vandenberg* (a former missile tracking ship intentionally sunk in 2009 off Key West, lower Florida Keys; 24°27.027′N, 081°43.991′W; sampled as FK-1068 in May 2014 and as FK-1148 in January 2016). Specimen collecting in the protected waters of the Florida Keys was conducted under Florida Keys National Marine Sanctuary Research Permit FKNMS-2013-105 to RB. These collecting events were part of a preliminary qualitative investigation of molluscan species on these shipwrecks, and no formal quantitative sampling was undertaken during these dives.

Whole molluscan specimens were compared to material in the collections of the Field Museum of Natural History (FMNH) that resulted from extensive prior and ongoing surveys in the Florida Keys National Marine Sanctuary (FKNMS; e.g., Bieler & Mikkelsen, 2004; Mikkelsen & Bieler, 2004; Mikkelsen & Bieler, 2007) and from studies on vermetid gastropods throughout the Atlantic Ocean (e.g., Bieler, 1995; Golding et al., 2014). Station designations beginning with “FK” refer to these ongoing biodiversity survey projects. FMNH number designations refer to specimen series lodged as voucher material and accessible in the Field Museum of Natural History (FMNH; database access under “invertebrates” at http://collections-zoology.fieldmuseum.org/). BivAToL numbers link to voucher material collected and previously cited in the context of the Bivalve Assembling-the-Tree-of-Life project (http://bivatol.org), with specimens likewise accessible via FMNH. Anatomical and morphological studies of the vermetid species followed the protocols outlined in Golding et al. (2014). Author/date references and acceptance of valid species follow the treatment in WoRMS (2017), except where specifically noted.

We used mitochondrial cytochrome oxidase 1 (COI) sequences in order to gauge whether the color morphs of the newly-described species likely represented intraspecific or species-level variation, and to see how much this new species differs at the COI locus from other *Thylacodes* species. We extracted the 530 nucleotide COI barcoding region from the complete mitochondrial genomes of three color morphs of *Thylacodes vandyensis* n. sp. (paratypes 1–3, see below). Similarly, the COI barcoding region was extracted from the complete mitochondrial genome for *Thylacodes decussatus* (Gmelin, 1791) (FMNH 327164, Belize). These complete genomes were generated on an Ion Torrent PGM with a 318 chip as part of a broader study of vermetid systematics in progress (Granados-Cifuentes et al., unpublished data). These COI sequences have been submitted to GenBank under the numbers KY586138, KY586139, KY586140, KY586141. In addition, we searched GenBank for COI sequences within the vermetid genus *Thylacodes* and the formerly used generic name *Serpulorbis*. We recovered 31 COI haplotypes of *Thylacodes variabilis* (Hadfield & Kay, 1972) (HM453681 –HM453711) from Hawaii, 1 haplotype of *Thylacodes* sp. from Australia (Colgan et al., 2007 [as *Serpulorbis* sp.]; AY296830), 1 haplotype of *Thylacodes* sp. from French Polynesia (Leray et al., 2013 as *Serpulorbis*; KC706891), 1 haplotype of *Thylacodes squamigerus* (Carpenter, 1857) from California, USA (Rawlings et al., 2010; HM174255), 1 haplotype of *Thylacodes medusae* Pilsbry, 1891 from Japan (Takano & Kano, 2014; AB930471), 1 haplotype of *Thylacodes “imbricatus”*^1^ 1The name *Thylacodes imbricatus* (Dunker, 1860) is preoccupied and not available. *Thylacodes adamsii* (Mörch, 1859) appears to be this species. See Bieler & Petit (2011: 70).from China (Zou, Li & Kong, 2011) as *Serpulorbis imbricata*; HQ834105) and 1 specimen of *Thylacodes* sp. from British Columbia, Canada (Layton, Martel & Hebert, 2014; KF643882). These sequences were aligned without indels using the program MAFFT (Katoh & Standley, 2013) in Mesquite (Maddison & Maddison, 2017). Absolute distances and uncorrected pairwise distances between sequences were generated in PAUP, version 4.0a151 (Swofford, 2002).

The electronic version of this article in Portable Document Format (PDF) will represent a published work according to the International Commission on Zoological Nomenclature (ICZN), and hence the new names contained in the electronic version are effectively published under that Code from the electronic edition alone. This published work and the nomenclatural acts it contains have been registered in ZooBank, the online registration system for the ICZN. The ZooBank LSIDs (Life Science Identifiers) can be resolved and the associated information viewed through any standard web browser by appending the LSID to the prefix http://zoobank.org/. The LSID for this publication is: urn:lsid:zoobank.org:pub:0535F741-EF90-49D7-B2DF-78E8E6EE8BC8. The online version of this work is archived and available from the following digital repositories: PeerJ, PubMed Central and CLOCKSS.

**Figure 3:**
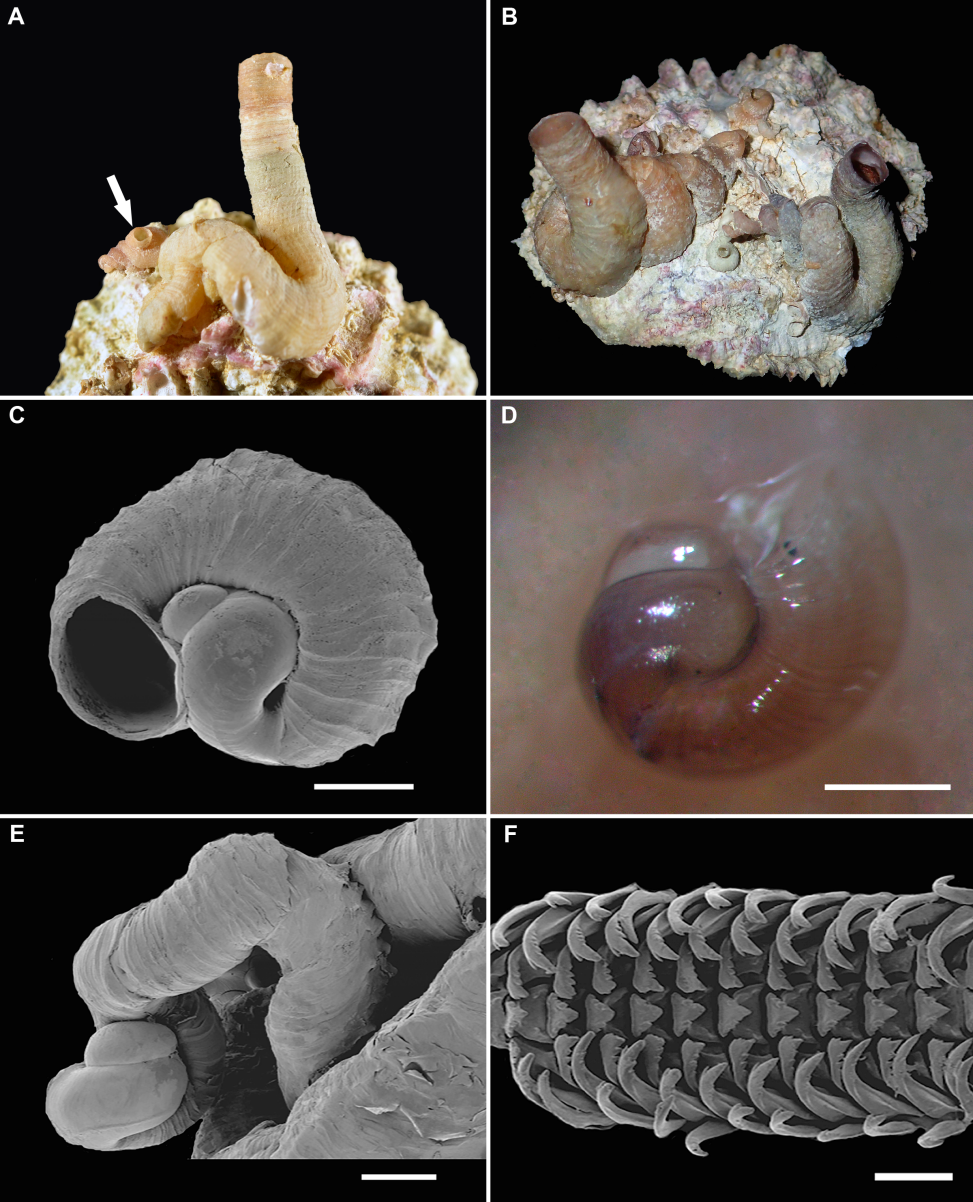
Shell morphology and radula of *Thylacodes vandyensis* n. sp. (all from collecting event FK-1148, 9 January 2016, about 29 m). (A) Holotype (FMNH 344615, large specimen, center) and juvenile paratype (FMNH 377465, arrow); outside diameter of holotype feeding tube at aperture 5.0 mm. (B) Group of paratypes on valve of *Hyotissa mcgintyi*, showing range of shell colors, after removal of sponge covering (FMNH 344619; length of bivalve shell, 41 mm). (C) Juvenile specimen removed from adult shell on which it had settled; note largely exposed protoconch (with comparatively weak spiral rib) being encircled by first teleoconch whorl (SEM; scale = 500 µm; FMNH 344614). (D) Living juvenile, freshly settled on adult female’s shell; note transverse shell ribbing, lack of orange and black body pigment at this stage, and black eye spots (FMNH 344614; scale = 500 µm). (E) Protoconch and early whorls of adult specimen; note the strongly developed rib on protoconch, the loose early teleoconch coiling, and the finely scalloped sculpture (SEM; FMNH 344614; scale = 500 µm). (F) Portion of adult radular ribbon, orange morph (SEM; FMNH 344614; scale = 200 µm).

## Results

### Non-native Bivalvia in the artificial reefs of the Florida Keys

The giant foam oyster, *Hyotissa hyotis* (Linnaeus, 1758)—also known as the giant honeycomb or giant coxcomb oyster, was the first report of a non-native molluscan species in the Florida Keys from shipwrecks, as discussed by Bieler et al. (2004) and Mikkelsen & Bieler (2007); Fig. 2B. This large-shelled Indo-Pacific species had frequently been confused with the presumably native foam oyster *Hyotissa mcgintyi* (Harry, 1985) [see discussion below and Fig. 2C], and Bieler et al. (2004) established that prior western Atlantic records of *H. hyotis* were in fact based on *H. mcgintyi* specimens. *Hyotissa mcgintyi* is widely distributed in the natural reef throughout the Florida Keys (e.g., in the Coffins Patch Sanctuary Preservation area in the middle Keys [FMNH 317472/FK-625], at Looe Key Reef in the middle Keys [FMNH 333460/FK-987], and off the Dry Tortugas [FMNH 317475/FK-606]. It also is a very common species on the investigated wrecks in all regions where it covers much of the available surface and acts as substratum for many other fouling organisms (e.g., *Spiegel Grove*, FMNH 335061/BivAToL-444/FK-1026, see ultrastructural sperm study in Healy, Mikkelsen & Bieler, 2015; *Benwood*, FMNH 335081/FK-1027; *Thunderbolt*, FMNH 302069/FK-717, see Figs. 83–94 and anatomical treatment in Simone, Mikkelsen & Bieler, 2015; *Adolphus Busch*, FMNH 333457/BivAToL-275/FK-908, see Figs. 2B, 8 and morphological and molecular analyses in Bieler et al., 2014a and Bieler et al., 2014b and *Vandenberg*, FMNH 341987/FK-1068).

A verified specimen of *H. hyotis*^2^ 2Huber (2010) distinguished two nominal species in this complex that previously had been considered synonyms, *Hyotissa hyotis* (Linnaeus, 1758) and *H. sinensis* (Gmelin, 1791). Available anatomical and molecular data are insufficient for addressing species delineations within the *H. hyotis* complex at this time.(FMNH 302010; for illustrations see Bieler & Mikkelsen, 2004: Figs. 3 and 4, and Mikkelsen & Bieler, 2007: 119) was first found in the Florida Keys in August of 2003 in about 30 m depth on the steel wreck of the lightning-strike research vessel *Thunderbolt*, off Marathon in the middle Florida Keys (FK-717). The 60-meter-long ship was intentionally sunk in 1986 as part of the Florida Keys Artificial Reef Association project and now lies intact on a sand bottom in about 37 m of water. An earlier unpublished record of another adult shell retrieved in 2001 north of the Florida Keys (off West Palm Beach; G Paulay, pers. comm. cited in Bieler *et al.*, 2004) likewise came from about 30 m water depth. Since then, this species has also been found in the upper Keys on the wreck of the *Spiegel Grove* (FMNH 335064/FK-1026; August 2012, in 31 m) and in the lower Keys on the wreck of the *Adolphus Busch* (FMNH 332211/FK-991, November 2011, in 28 m), both first reported herein (Fig. 2B). The specimens from the *Thunderbolt* and *Spiegel Grove* wrecks became the basis for extensive anatomical studies of *H. hyotis* by Simone, Mikkelsen & Bieler (2015: Figs. 95–104).

It thus appears that non-native *H. hyotis* has established a population in the Florida Keys. This is in contrast to another large-shelled Indo-Pacific bivalve, the black-lipped pearl oyster *Pinctada margaritifera* (Linnaeus, 1758), specimens of which have been recorded at several locales off the South Florida coast since the early 1990s (e.g., Chesler, 1994; Carlton & Ruckelshaus, 1997), but apparently did not spread further or move to artificial reefs of the region (R Bieler, pers. obs.). Likewise, the non-native mussel species *Perna perna* (Linnaeus, 1758) and *P. viridis* (Linnaeus, 1758) that have been recorded from the Gulf of Mexico (e.g., Ingrao, Mikkelsen & Hicks, 2001) have not yet been reported from the Florida Keys.

### Potentially non-native Gastropoda in the artificial reefs of the Florida Keys

*Cyclothyca pacei* (Petuch, 1987) is a small-shelled, cap-shaped gastropod species first reported from shallow-water in Biscayne Bay, Florida. Originally described as a member of the caenogastropod family Capulidae, the species is now recognized as a member of the heterobranch (pyramidelloidean) family Amathinidae that lives as an ectosymbiont on living specimens of pectinid (e.g., *Caribachlamys*) and spondylid (*Spondylus* spp.) bivalves. It is not uncommonly collected off Southeastern Florida (Petuch, 1987; Lee, 2011) and is now well established on the artificial reefs off the Florida Keys, herein first reported from the wrecks of the *Spiegel Grove* (FMNH 328533/FK-1026, August 2012), *Thunderbolt* (FMNH 301979/FK-717, August 2003), and *Vandenberg* (FMNH 328533/FK-1068, May 2014); Fig. 2D. The *Thunderbolt* specimens formed the basis for an anatomical analysis of this species by Simone (2011).

Because *C. pacei* associates with scallop species that are very popular with shell collectors, Lee (2011) suggested that the relatively recent discovery of the nominal species might indicate a recent arrival to the Florida fauna. Lee also proposed that it might be an ecomorph of the larger-shelled Pacific Ocean species *Amathina tricarinata* (Linnaeus, 1767), and compared it also to the eastern Pacific species *C. corrugata* Stearns, 1891. The latter species, originally described from the Pacific coast of Nicaragua and only recently recollected (Stearns, 1891; Garcia, 1996) might indeed be conspecific. Unfortunately no anatomical and/or molecular samples are available for the Pacific form; however, DNA samples have been vouchered, and gene sequences generated, from Florida specimens. We consider *C. pacei/corrugata* as a likely non-native species of Pacific origin with extensive populations on Florida’s artificial reefs. It has yet to be reported from shallower water and/or natural habitats in the Florida Keys.

In the following, we describe an additional gastropod, a new species of the caenogastropod family Vermetidae, from the artificial reefs. To date it is known only from the wreck of the *Vandenberg*:

**Table:** 

**Figure 4:**
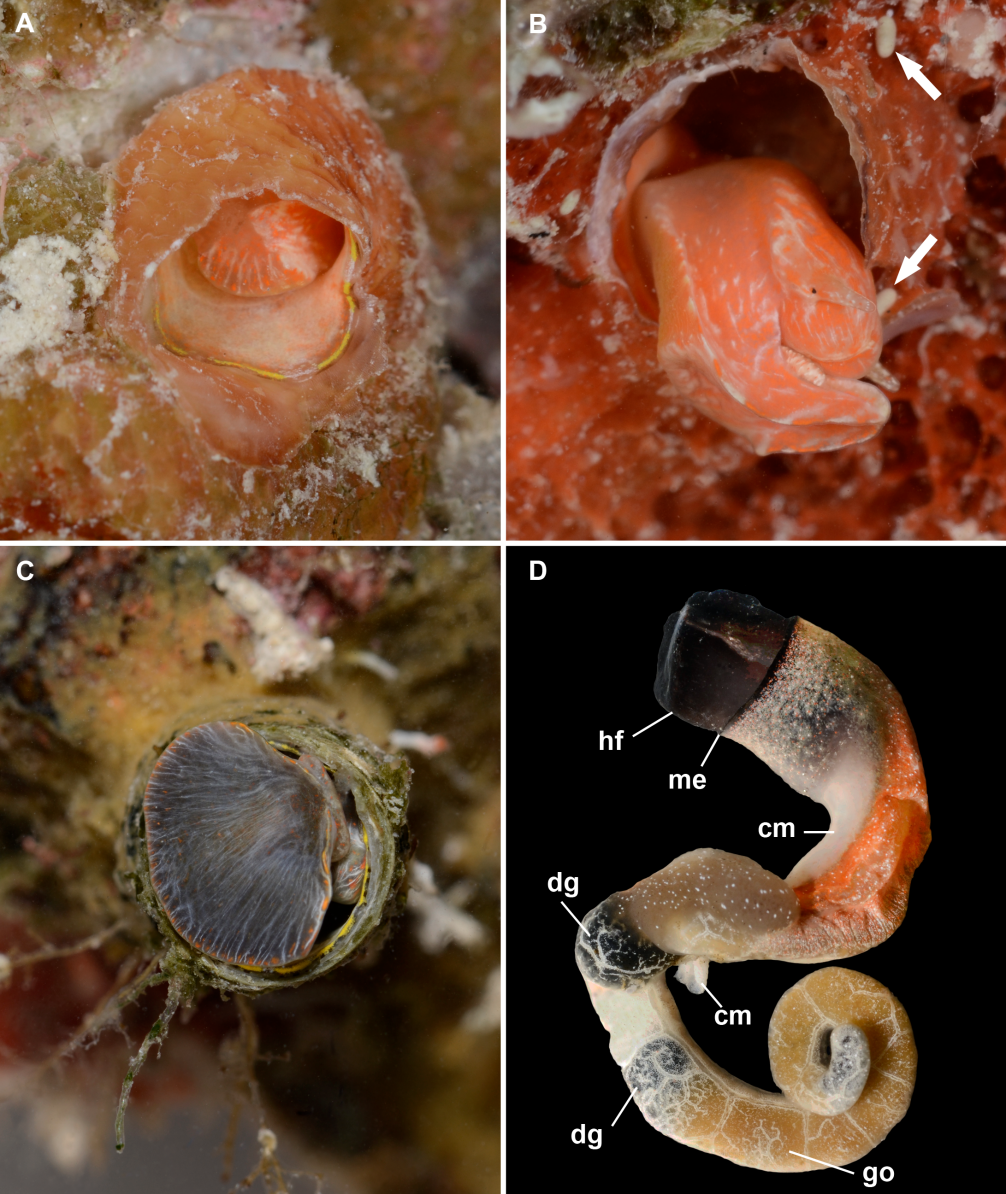
External morphology and living coloration of *Thylacodes vandyensis* n. sp. (A) (paratype 1) Orange morph, with yellow pattern on mantle edge; oblique view into shell aperture with animal slightly withdrawn but keeping the mantle extended; on *Hyotissa mcgintyi* (diameter of shell aperture, 4.5 mm). (B) (paratype 2) Orange morph, without yellow pattern on mantle edge; right lateral view of extended head-foot; note upper set of cephalic tentacles (with one black eye visible) and lower set of pedal tentacles; elongated-ovoid fecal pallets (arrows) near the extremely thin wall of the feeding tube; on *Spondylus americanus* densely covered by a red encrusting sponge (*Mycale* sp.) (diameter of shell aperture, 4.5 mm). (C) (paratype 3) Grey morph, with yellow pattern on mantle edge; animal slightly protruding from feeding tube, showing entry to mantle cavity; on *Hyotissa mcgintyi* (diameter of shell aperture, 6.5 mm). (D) Grey morph, without yellow pattern on mantle edge (me); whole male animal removed from shell; note near-black head-foot (hf), orange-lined extent of mantle cavity, white columellar muscle (cm), olive-black digestive gland (dg), and tan gonad (go) (FMNH 344614; from about 29 m depth, September 2016; same animal as in Fig. 5B; largest diameter, at mantle edge, 6.0 mm).

**Figure 5:**
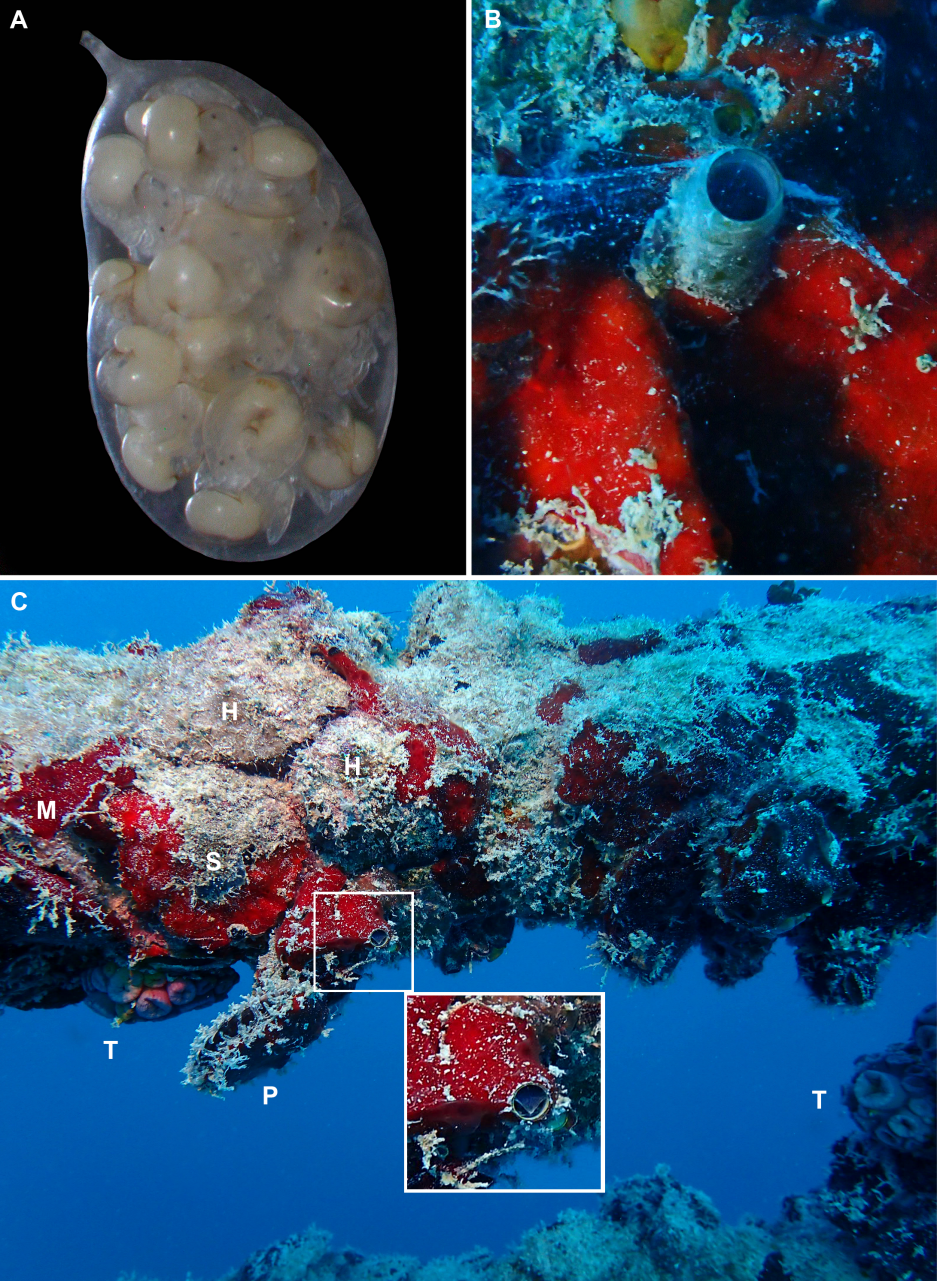
Egg capsule, mucous net feeding, and fouling community of *Thylacodes vandyensis* n. sp.; all in or from about 29 m depth, September 2016. (A) Egg capsule containing 16 juveniles very close to hatching; note short off-center capsule stalk (length of capsule without stalk, 4.1 mm; FMNH 344614). (B) Solitary animal (grey morph) in the process of mucous net feeding, on *Hyotissa mcgintyi* overgrown by a red encrusting sponge (*Mycale* sp.); same animal as in Fig. 4D (photographed in September 2016 on the side of the ship’s superstructure; diameter of vermetid tube opening approximately 5.5 mm; FMNH 344614). (C) Portion of the heavily fouled deck rail of the wreck in partial artificial light; with grey morphs of *T. vandyensis* (two specimens enlarged in insert, with right animal showing yellow mantle margin pattern), specimens of the orange cub coral *Tubastraea concinna* [T], and the bivalves *Hyotissa mcgintyi* [H], *Pinctada imbricata* [P], and *Spondylus ambiguus* [S]. Densely covered by red encrusting sponge (*Mycale* sp. [M]); diameter of vermetid tube openings approximately 5.5 mm. [All photographs by the senior author].

***Thylacodes vandyensis*Bieler, Rawlings & Collins n. sp.**
Figs. 2A, 3A–3F, 4A–4D, 5A–5C.

*Type locality*: Wreck of the *USNS General Hoyt S. Vandenberg*, between Western Sambo Reef and Sand Key, about 11 km off Key West, Florida, USA, Atlantic Ocean24°27.164′N, 081°43.594′W; in about 29 m depth (see Fig. 1).

*Material examined:* All specimens from type locality. Paratypes 1–3 from collecting event FK-1068 (26 May 2014), all others, including holotype, from collecting event FK-1148 (9 January 2016): **Holotype** (Fig. 3A): FMNH 344615: wholly dry-preserved specimen (greatest length of attached shell mass 18.5 mm, outside diameter of feeding tube aperture 5.0 mm) on substratum (shell of *Hyotissa mcgintyi*), with juvenile **Paratype-4** (FMNH 377465, greatest length of entire shell 6.6 mm). **Additional paratypes** (not numbered; multiple specimens, dry and in various preservation fluids): FMNH 344619 (with specimens of Fig. 3B), 344614 (with specimens of Figs. 3C–3F, 4D), 344616, 344617, 357557, 357587, 376313, 376314). **Paratype-1** (Fig. 4A), FMNH 328529: 1 specimen removed from shell of *Hyotissa mcgintyi* (body in 80% ethanol, tissue samples, dry shell voucher). **Paratype-2** (Fig. 4B), FMNH 328530: 1 specimen removed from shell of *Spondylus americanus* (body in 80% ethanol, tissue samples, dry shell voucher). **Paratype-3** (Fig. 4C), FMNH 328531: 1 specimen removed from shell of *Hyotissa mcgintyi* (body in 80% ethanol, tissue samples, dry shell voucher). **Paratype-5** deposited in Museo Nacional de Ciencias Naturales, Madrid, Spain, MNCN 15.05/200050 (1 specimen wholly preserved in 80% ethanol; ex FMNH 344616).

Description:

*Teleoconch* (Figs. 2A, 3A–3C, 5B): Largest length of attached individual adult shell mass usually 20–25 mm; length of standing portion of adult tube above attached shell mass 9–25 mm; largest diameter of attached shell whorl about 6–7 mm; diameter at apertural opening of feeding tube in adults about 4.5–6.5 mm. Not entrenching into the substratum; with convoluted, irregular growth, often changing direction and wrapping back over earlier coils, with sharp elbow-like bends that frequently show remnants of earlier feeding tubes; attached adult whorls often widened into an attachment flange; earlier whorls abandoned and closed off by shell septa, with body residing in final 2–3 attached whorls; early whorls remaining narrow for several, usually loose, coils and with growth lines and/or densely spaced transverse ribbing (Figs. 3C–3E); later whorls with faint to well-expressed longitudinal ribbing; when well-expressed this sculpture consisting of about 6 larger ribs visible on the surface of attached whorls, with 1–3 weaker ones in the interspaces; ribs forming knobs and fine scales at intersections with growth marks; last part of whorls usually as an erect feeding tube, narrower and thinner-walled than the preceding attached part and lacking longitudinal sculpture; terminal feeding tube portion often further narrowed; thin apertural edge often very finely scalloped, resulting in corresponding microscopic scale pattern on the shells. Inside wall of shell tube macroscopically smooth, without columellar laminae.

Color overall light to reddish tan, with knobby parts of ribs, especially in the flange area of thicker-shelled adult whorls, lighter.

*Protoconch* (Figs. 3C–3E): About 1.5 rapidly expanding bulbous whorls, forming narrow umbilicus; lower part of whorl with weakly (Fig. 3C) to strongly (Fig. 3E) developed spiral rib; surface smooth except for weak wrinkles near spiral rib (Fig. 3C) and densely spaced, microscopic, spiral striae, most visible near apertural lip; light reddish tan, initial part lighter colored.

*Operculum* absent.

*Radula* (Fig. 3F): Length of adult radular ribbon about 3.1 mm; up to 30 rows. Taenioglossate; rachidian and lateral teeth with 3 (sometimes 4) cusps on either side of strong central cusp; inner marginal tooth with long and strong main cusp, 3–4 small cusps on outer side, and single longer cusp on inner side; slender outer marginal tooth with single cusp on inner side. No difference noted between grey and orange morphs.

*External morphology and soft-body coloration* (Figs. 4A–4D): Comparatively stocky vermetid body, with plug-like head-foot complex and very short (rarely more than 3 mm long) free end of columellar muscle. Color in life: Polychromatic:  Orange morph (Figs. 4A and 4B): Pedal disk with overall deep orange tissue coloration, with white lines radiating from a position near the mouth and extending and divaricating toward the pedal side; white line pattern usually strongest near pedal-side margin, with concentrated areas of orange pigment between them at the edge, resulting in a more or less regular alternating orange-white pattern. Head-foot areas entirely without black pigment (except eye spots), with mottled pattern of white and orange; both pairs of tentacles, but especially pedal tentacles, with small white pigment granules; inside mantle salmon colored.  Grey morph (Figs. 4C, 4D; 5B, 5C): As above, but the orange within-tissue base coloration covered and/or mostly (but never completely) replaced by near-black; the resulting combination of this dark tissue with the orange granules and whitish lines near the surface imparting overall olive-grey (in extreme cases, velvety black) appearance; inside mantle of these animals off-white.  Both color morphs usually with the mantle edge lined with alternating pattern of yolk-yellow and narrower translucent white pattern (with yellow blotches in the dark form often showing fine black pigment on outside margin; Figs. 4A, 4C), but some specimen of both color forms lacking the yolk-yellow pigment entirely (Figs. 4B and 4D). Mantle cavity region usually intensely orange, even in specimens with black head-foot (Fig. 4D). Ethanol-preserved specimens (80%) fade to overall tan, with only the black head-foot pigment remaining.

*Pallial and reproductive anatomy* (Figs. 4D and 5A): Gill with up to 120 orange leaflets, about 22 per mm in the adult mid-gill. Reproductive females with anterior mantle slit to accommodate stalk of egg capsules, which are brooded in the mantle cavity and attached to the inside wall of the shell tube.

*Development* (Fig. 5A): 3–5 short-stalked whitish-translucent egg capsules per female observed herein, about 4.0–4.2 mm in length close to hatching; 12–16 embryos per capsule. Hatchlings with translucent-tan shells; brown stains in suture and umbilical area; crawling with vela fully resorbed.

*Habitat and ecology* (Figs. 2A; 5B, 5C): Affixed to substratum (mostly shells of living and dead *Spondylus americanus* Hermann, 1871 and *Hyotissa mcgintyi*), in current-exposed settings on the wreck’s superstructure, hull, and rails shared with the bivalves *Arca zebra* (Swainson, 1833), *Acar* sp. [“*domingensis*” of authors], *Barbatia candida* (Helbling, 1779) [all Arcidae]; *Pinctada imbricata* Röding, 1798 [Pteriidae]; *Isognomon radiatus* (Anton, 1838) [Isognomonidae]; *Ctenoides mitis* (Lamarck, 1807) [Limidae]; *Lindapecten muscosus* (Wood, 1828) [Pectinidae]; *Anomia* sp. [Anomiidae]; *Dendostrea frons* (Linnaeus, 1758) [Ostreidae]; *Spondylus ambiguus* Chenu, 1844^3^ 3*Spondylus tenuis* Schreibers, 1793 would have priority if that name is considered valid. The taxonomic issue was discussed by Simone, Mikkelsen & Bieler (2015: 57).; Spondylidae]; *Gastrochaena* sp. [Gastrochaenidae]; *Chama macerophylla* Gmelin, 1791, and *Chama* sp. [Chamidae]; the orange tube coral *Tubastraea coccinea*; the red encrusting sponge *Mycale* sp. (Demospongiae: Poecilosclerida: Mycalidae); and various other invertebrates yet to be identified (voucher specimens of listed mollusks deposited in FMNH). Adult shells found singly, in pairs, or in clusters; occasionally with settled juveniles on the outside shell tube. Active suspension feeder by mucous web (Fig. 5B).

*Density*: First discovered, in small numbers, in about 29 m depth on the wreck of the *USNS Vandenberg* off Key West in in May of 2014, exactly five years after the vessel was sunk at the site. About 19 months later, in January 2016, the population had spread over much of the current-exposed parts of the wreck in the 30 m depth range. Short dive durations did not allow for extensive density measurement to date, but in the apparently most densely settled areas on the deck’s rails, 9 adults (plus juveniles) were recorded from a single 5 × 5 cm surface area.

*Distribution*: Currently only known from the type locality.

*Etymology*: *vandyensis*, -*e*: named for the wreck of the “Vandy,” the nickname given by the SCUBA diving community to the *USNS General Hoyt S. Vandenberg*, a retired naval vessel sunk to serve as an artificial reef off the lower Florida Keys.

*Comparative and taxonomic remarks*: Because of the serpulid-worm-like irregular shell morphology of vermetids that defies classification attempts based solely on adult shell characters, and the unusually complex historic literature of this group that is commingled with that of polychaete worms and various other uncoiling molluscan groups (e.g., Bieler, 1996), vermetid taxonomy needs to be based on multiple suites of characters. Among others, these include aspects of the larval shell, the coloration of the head-foot and mantle edge, the morphology and organization of the egg capsules, and—when present—the morphology of the operculum (e.g., Golding et al., 2014). Whereas most vermetids have a more-or-less elaborate operculum allowing for partial or complete closure of the aperture, at least one clade does not. This non-operculate vermetid group, to which *Thylacodes vandyensis* n. sp. belongs, has been known in the literature largely under the generic name *Serpulorbis* Sasso, 1827, which was shown to be a synonym of *Thylacodes* Guettard, 1770 by Bieler & Petit (2010). Five western Atlantic species were traditionally placed in *Serpulorbis/Thylacodes* (Rosenberg, 2009), two of which (*Serpulorbis birugosus* Weisbord, 1962 and *S. catella* Weisbord, 1962) are now recognized to be based on polychaete worm tubes (Bieler & Petit, 2011). Two others, *T. decussatus* (Gmelin, 1791) and *T. riisei* (Mörch, 1862), differ from *T. vandyensis* n. sp. in having shells with much more developed longitudinal ribs and a pedal coloration lacking white streaks (R Bieler, pers. obs.). *Thylacodes squamolineatus* (Petuch, 2002), a poorly known taxon originally described, as *Serpulorbis*, from a single tube fragment collected in the Bimini Chain, Bahamas, has a much larger shell with an apertural diameter exceeding 10 mm and also a more strongly developed longitudinal sculpture on its (based on the limited available material, apparently white) shell. The shell of *T. vandyensis* n. sp. is superficially similar to those of species of *Petaloconchus* (e.g., *P. varians* [d’Orbigny, 1839]) occurring in shallower waters in Florida, but members of that group differ in many morphological features including the presence of opercula and columellar shell laminae.

Species of *Thylacodes* often are polychromatic in their head-foot tissue appearance (R Bieler, pers. obs.), but usually share a specific pattern of the mantle edge. The facultative lack of black and/or yellow pigment in parts of this population initially suggested the presence of more than one species (but see molecular markers, below). We are aware of additional, currently unnamed, vermetid species in the western Atlantic that lack opercula and would thus traditionally be placed in *Serpulorbis/Thylacodes*. None of these match the combination of shell characters and, where known, head-foot coloration of the newly described species.

In the Mediterranean and eastern Atlantic Ocean, the much larger-shelled *Thylacodes arenarius* (Linnaeus, 1758) reaches a shell aperture diameter of 11–15 mm and its head-foot and mantle coloration is characterized by a different pattern of a usually wine-red base color with solid-white streaks and patches (R Bieler, pers. obs.).

Our research team is very familiar with the Atlantic worm-snail fauna and we have not encountered this relatively large-shelled vermetid species anywhere in the field or in extensively studied museum collections. By contrast, we are aware from preliminary studies that large numbers of vermetid species in the Indo-Pacific have remained undescribed. We therefore consider it likely that this species is a recent arrival from Pacific waters, parallel to the case of the foam oyster *Hyotissa hyotis*. Comparative morphological data for Indo-Pacific members of *Thylacodes* were compiled by Kelly (2007, Table 1; as *Serpulorbis*); none of these match the characters here observed for *T. vandyensis* n. sp. Although there was great variation in shell sculpture and soft-body color and patterning in *Thylacodes vandyensis* n. sp., the amount of COI variation was modest among the three color morphs sampled. Sequence divergence ranged from 0.38% to 0.94%, which is well within the typical range of intraspecific variation for this locus (Hebert et al., 2003). *Thylacodes vandyensis* n. sp. COI sequences were most similar to those reported for *Thylacodes medusae* sampled from off Misaki in Kanagawa Prefecture, Japan (Takano & Kano, 2014) , differing by 17.7 to 18.1%, and those of *Thylacodes* “*imbricatus*” [*adamsii*] from China (Zou, Li & Kong, 2011), differing by 20.3 to 20.8%. Both of these species are much larger- and sturdier-shelled, with adult apertural shell diameters of *T. medusae* (e.g., FMNH 138381, syntypes, Japan) reaching at least twice those of *T. vandyensis* n. sp.

## Discussion

With nearly 400 bivalve species and some 1,300 gastropod species currently recognized as occurring in the Florida Keys (Bieler & Mikkelsen, 2004; R Bieler & PM Mikkelsen, 2017, unpublished data; Mikkelsen & Bieler, 2000; Mikkelsen & Bieler, 2004; Mikkelsen & Bieler, 2007), the majority of them small-bodied and cryptic, distinguishing between native and non-native fauna is difficult. Geographic boundaries in the marine environment are literally fluid, and an “unusual occurrence” might be the result of a mere expansion of a nearby distribution, an extension of a deeper-water taxon into shallower depths, or part of the natural range of a previously overlooked local species. Recognition of non-indigenous species in an area requires baseline data on natural species distribution, taxonomic expertise to reliably identify species, and monitoring efforts to recognize unusual occurrences. Taxonomic identification at the species level warrants specialized morphological knowledge and, ideally, verification with molecular data. The appearance of an unusual species on a monitored artificial reef may be a minor extension of a native species or it can be the first sign of an invasive event. Often, the investigator needs to look at museum-based specimens of prior decades to establish historic presence or absence in the region, as was done in the studies that first reported *Tubastraea coccinea* and *Hyotissa hyotis* as recent non-native arrivals, as well as with the newly reported vermetid species herein. Whereas some of the molluscan species that commonly occur on the deeper artificial reefs, such as *Arca zebra* and *Spondylus americanus*, clearly are members of the Floridian/Caribbean fauna and merely extended their populations to the newly available substratum, others will require additional investigation. In addition to the case of *Cyclothyca pacei* described above, these also include the previously mentioned foam oyster *Hyotissa mcgintyi*, the most common oyster on the wrecks of the Florida Keys (see Fig. 2C). Originally named as *Ostrea thomasi* McLean, 1941 and described from a single specimen dredged off Palm Beach, Florida, it was later renamed as *Parahyotissa mcgintyi* Harry, 1985 because of homonymy with the unrelated *Ostrea sellaeformis* var. *thomasii* Glenn, 1904 (Harry, 1985). In southeast Florida, it was described as “uncommon: below low tide and on offshore wrecks” by Abbott (1974: 457, misidentified as *Pycnodonte hyotis*). *H. mcgintyi* is well known from Gulf of Mexico oil and gas platforms where it is one of the most abundant oysters (Harry, 1986) and is “abundant on the offshore coral reefs off Texas and locally almost reef forming on Miocene shale outcrops” according to Odé (1980: 49, misidentified as *Pycnodonte hyotis*). In the Florida Keys, it now is a very common species both in the natural barrier reef and on the artificial reefs. How long *Hyotissa mcgintyi* has been a member of the western Atlantic fauna is not yet clear; it has been considered conspecific with an eastern Atlantic oyster by Huber (2010 under the forgotten name “*Parahyotissa rosea* (Gmelin, 1791)”) and may in fact be a fairly recent arrival itself that spread quickly on artificial reefs in the Gulf of Mexico and the Florida Keys.

In the cases of the introduced bivalves reported above, ballast water and vessel hull fouling were considered the most likely vectors of introduction (e.g., Bieler *et al.*, 2004). As sessile members of the fouling community, vermetids are particularly capable of dispersal by rafting and individual species have been tagged as potentially invasive in the Pacific Ocean (Bieler in Carlton, 1999: 449; *Vermetus alii* Hadfield & Kay, 1972, now recognized as *Eualetes tulipa* [Rousseau in Chenu, 1843]) or with a high potential to be invasive (Strathmann & Strathmann, 2006). In contrast to accidentally sunk ships, the deliberately scuttled vessels of the Florida Keys are less likely to have carried invasive species at the time of their sinking, as cleaning of the vessels from environmentally harmful substances and potentially invasive species have been mandatory parts of the preparation and permitting process in recent years (EPA, 2006). Vessel cleaning has not always been successful, however, such as in the failed attempt to kill the entire fouling community on the decommissioned battleship, *USS Missouri*, through prolonged immersion in brackish water prior to its transfer to Pearl Harbor, Hawaii. This resulted in the spawning and successful recruitment of the Mediterranean mussel (*Mytilus galloprovincialis* Lamarck, 1819) to surfaces within Pearl Harbor (including the ballast tank of a US Navy submarine) within days to weeks of the ship’s arrival (Apte et al., 2000).

Challenges for a colonizer include (1) the initial dispersal to the site and (2) the successful growth and maintenance of a viable population. Species with long-lived swimming larvae can reach newly available sites comparatively quickly. It is therefore no surprise that among the few predatory gastropods found during this study on the Vandenberg wreck are members of the Ranellidae (*Monoplex nicobaricus* [Röding, 1798], FMNH 342016, 376315; *Monoplex pilearis* [Linnaeus, 1758], FMNH 377029) and Bursidae (*Bursa rhodostoma* [G. B. Sowerby II, 1835], FMNH 377028), groups known to have long-range, teleplanic larvae (e.g., Scheltema, 1971; Scheltema, 1972). However, with the often very strong current at these artificial wreck sites, a mandatory long larval phase would not support the rapid buildup of a local population from a few founders (or even a single gravid female). Vermetids are known to show a range of reproductive modes, with some species releasing swimming larvae that need to feed and grow in the plankton, whereas in others the females brood the developing embryos and larvae in the mantle cavity until crawling juveniles emerge. *Thylacodes vandyensis* n. sp. belongs to the latter group with crawl-away juveniles hatching from brooded egg capsules. As discussed by Johannesson (1988), direct-developing species can often have much wider geographic distributions than would be predicted based on the limited dispersal abilities of juveniles and adults. Despite the absence of a planktonic larval form, these species have colonized isolated locations such as oceanic islands, often far from presumed source populations (e.g., Johannesson, 1988; O’ Foighil, 1989), likely through rafting events or hitchhiking as part of fouling communities (Winston, 2012). For vermetids such as *Thylacodes vandyensis* n. sp., which form permanent attachments to both natural and artificial surfaces in the marine environment, such a lifestyle predisposes them to transport on vessel hulls and other drifting substrates and potentially to this type of introduction to new habitats. Vermetid hatchlings have been observed to produce mucous threads that are then transported by water currents (Strathmann & Strathmann, 2006) which can provide a mechanism by which these juvenile vermetids move within a habitat but also potentially colonize new habitats such as artificial reefs. Once present in a new location, founder populations consisting of direct developing species can also experience rapid rates of population growth associated with the low mobility of their life-history stages which ensure high encounter frequencies between individuals for mating (Johannesson, 1988). Traits associated with vermetids including intracapsular development within the protective confines of their shell tube, long term sperm storage, and hermaphroditism in some species (Calvo & Templado, 2005; Strathmann & Strathmann, 2006) can also be viewed as traits enhancing rapid population growth of founding populations.

The creation of novel, initially-empty habitats such as artificial reefs may have important consequences for arriving non-native species and adjacent natural populations. These new habitats may act as beach heads or stepping-stones for non-native species (Simberloff, 1997; Glasby et al., 2007). Although there is a steady flow of ship traffic in the Florida Keys, these artificial reefs may act as permanent way-stations for arriving non-natives, providing nurseries within which populations may grow in an environment with reduced competition compared to native habitats (Tyrell & Byers, 2007), and then, in some cases, spread to natural habitats (Bulleri & Airoldi, 2005; Glasby et al., 2007). Hard substrates, such as those found on sunken vessels, have, in particular, been implicated in the establishment and spread of non-native populations (Ruiz et al., 2009; Sheehy & Vik, 2010), and, since non-native species are most often transported on artificial substrates such as ship’s hulls and ballast tanks, they may be pre-adapted to life on sunken vessels. In the Florida Keys, ships visiting these artificial reefs for recreational use may facilitate the further spread of fouling organisms (Sheehy & Vik, 2010). Of course, many native species also become established in these new habitats, but the fauna of artificial reefs may remain distinct from surrounding natural reefs (Carvalho et al., 2013), in some cases on the scale of a century or more (Perkol-Finkel, Shashar & Benayahu, 2006). All of this suggests that artificial reefs are one of the best places in the marine environment to monitor regularly for the presence of newly arrived non-native species (Ruiz et al., 2009; Peirano, 2013) and identification of new and potentially problematic non-native species at the earliest stages of their introduction increases the chances of effective control or eradication.

Although vermetids are common constituents of coral reef habitats, there is growing evidence of negative interactions between vermetids and neighboring coral species. As recently summarized by Shima, Phillips & Osenberg (2016), vermetids can cause growth anomalies in corals and are known to substantially reduce coral growth (Colgan, 1985; Zvuloni, Armoza-Zvuloni & Loya, 2008; Shima, Osenberg & Stier, 2010; Stier et al., 2010; Shima, Phillips & Osenberg, 2013) and might be able to shift coral species composition in a region (Shima, Osenberg & Stier, 2010). Colgan (1985) suggested that the vermetid mucous feeding web can inhibit its coral host’s growth in four ways: by reducing water circulation, by attenuating light, by intercepting food, or by irritating the coral’s surface. Indeed, the presence of vermetids and their interaction with coral tissue also appear to trigger pathogenic processes or increase the rate of coral disease transmissions. Misinterpreting the feeding ecology of the suspension feeding vermetids as “corallivorous” (Al-Hammady & Mohamed, 2016: 5), the authors claimed that the most commonly encountered coral disease in their Red Sea study area, atramentous necrosis, “is always associated with Vermetidae^4^ 4The supposed corallivorous vermetid species was not identified in the article, but from the photograph (Al-Hammady & Mohamed, 2016: Fig. 3E) it appears to be *Ceraesignum maximum* (G.B. Sowerby I, 1825), a species well described for its suspension feeding (e.g., Kappner, Al-Moghrabi & Richter, 2000; as *Dendropoma maxima*).predation” (2016: 1). In fact, the deleterious effect on the coral tissue surrounding the vermetid feeding tube (including tissue necrosis) has been shown to stem from bioactive metabolites generated by the vermetids and included in their mucous feeding nets (Klöppel et al., 2013). The bioactive substances seem to have at least two different effects beneficial to the vermetids: fish avoid the feeding webs (Klöppel et al., 2013; R Bieler, pers. obs.) and coral growth around the settled vermetid is reduced or even stopped. As a consequence, the introduction of a new vermetid species to the natural reefs of the Florida Keys may have deleterious effects on coral growth and survival.

## Conclusions

The artificial reefs of the Florida Keys attract invertebrate species that are recognized as non-native (the orange tube coral *Tubastraea coccinea* and the giant foam oyster *Hyotissa hyotis*, both herein reported from new localities). Some other molluscan species common on these wrecks may likewise be of extra-regional origin (*Hyotis mcgintyi* and *Cyclothyca pacei*). The newly described vermetid species, whatever its point of origin, is a new arrival to the Florida Keys. *Thylacodes vandyensis* n. sp. is perhaps atypical of non-native arrivals in that it broods its young in egg capsules that hatch crawl-away juveniles. While this would seem to lower the chances of initial arrival, it would increase its chances of establishment on a sunken vessel in strong currents. A potential transfer of any newly arrived species from the artificial reefs into the nearby barrier reef system would be a valid concern in any case. In this particular situation, involving a member of Vermetidae, the situation is of special interest and concern as vermetid-coral interactions are known to be potentially harmful to hard coral growth and survival. Vermetids have also been shown to function as intermediate hosts for blood flukes (family Spirochiidae) that parasitize the loggerhead turtle, *Caretta caretta* (Linnaeus, 1758), see Cribb et al. (2017). Years of focused taxonomic study on the mollusks, access to historical museum collections, and regular resampling within the Florida Keys enabled these discoveries. It is likely that similar discoveries await in other taxonomic groups. A modest investment in regular “bioblitz” sampling with broad taxonomic expertise within sentinel habitats such as artificial reefs and marinas would enable much earlier recognition of new and potentially problematic non-native species, facilitating effective responses. Ongoing monitoring of the developing molluscan fauna on the artificial reefs of the Florida Keys will be a vital part of assuring natural reef health and providing the necessary evidence for implementation of control or eradication measures when new and potentially problematic non-native species are discovered.

## Additional Information and Declarations

### Field Study Permissions

The following information was supplied relating to field study approvals (i.e., approving body and any reference numbers): Specimen collecting in the protected waters of the Florida Keys was conducted under Florida Keys National Marine Sanctuary Research Permit FKNMS-2013-105 to RB.

### DNA Deposition

The following information was supplied regarding the deposition of DNA sequences: The COI sequences described here are accessible via GenBank accession numbers KY586138, KY586139, KY586140, KY586141.

### Data Availability

The following information was supplied regarding data availability: Core taxonomic and collection-event data for newly and previously collected mollusks cited in this study are available online through Field Museum of Natural History’s (FMNH) institutional collections database. The Invertebrates collection accession numbers are: 138381, 301979, 302010, 302069, 317472, 317475, 328529, 328530, 328531, 328533, 332211, 333457, 333460, 344618, 335061, 335064, 335081, 341987, 342016, 344614, 344615, 344616, 344617, 344619, 357557, 357587, 376313, 376314, 376315, 377028, 377029, 377465. http://collections-zoology.fieldmuseum.org/.

### New Species Registration

The following information was supplied regarding the registration of a newly described species: Publication LSID: urn:lsid:zoobank.org:pub:0535F741-EF90-49D7-B2DF-78E8E6EE8BC8; Species name: urn:lsid:zoobank.org:act:8719E699-CE0C-4A55-81DB-A627B35E31D3.

## References

[ref-1] Abbott RT (1974). American seashells: the marine mollusks of the Atlantic and Pacific Coasts of North America.

[ref-2] Akins L, Semmens C (2011). Final report, reef monitoring of the artificial reef Gen. Hoyt S Vandenberg, Key West, Florida, April 30, 2009 to July 19, 2010. http://www.reef.org/reef_files/Vandenberg_1year_finalreport_w_appendicies.pdf.

[ref-3] Al-Hammady MAM, Mohamed MH (2016). Distribution and disease prevalence of coral associated bacteria at some impacted Red Sea reefs. Journal of Biodiversity & Endangered Species.

[ref-4] Apte S, Holland BS, Godwin LS, Gardner JPA (2000). Jumping ship: a stepping stone event mediating transfer of a non-indigenous species via a potentially unsuitable environment. *Biological Invasions*.

[ref-5] Atchison AD (2005). Offshore oil and gas platforms as stepping-stones for expansion of coral communities: a molecular genetic analysis. MS Thesis.

[ref-6] Bieler R (1995). Vermetid gastropods from São Miguel, Azores: comparative anatomy, systematic position and biogeographic affiliation. Açoreana Supplement.

[ref-7] Bieler R (1996). Mörch’s worm-snail taxa (Caenogastropoda: Vermetidae, Siliquariidae, Turritellidae). American Malacological Bulletin.

[ref-8] Bieler R, Mikkelsen PM (2004). Marine bivalves of the Florida Keys: a qualitative faunal analysis based on original collections, museum holdings and literature data. Malacologia.

[ref-9] Bieler R, Mikkelsen PM, Collins TM, Glover EA, González VL, Graf DL, Harper EM, Healy J, Kawauchi GY, Sharma PP, Staubach S, Strong EE, Taylor JD, Tëmkin I, Zardus JD, Clark S, Guzmán A, McIntyre E, Sharp P, Giribet G (2014a). Investigating the bivalve tree of life—an exemplar-based approach combining molecular and novel morphological characters. Invertebrate Systematics.

[ref-10] Bieler R, Mikkelsen PM, Collins TM, Glover EA, González VL, Graf DL, Harper EM, Healy J, Kawauchi GY, Sharma PP, Staubach S, Strong EE, Taylor JD, Tëmkin I, Zardus JD, Clark S, Guzmán A, McIntyre E, Sharp P, Giribet G (2014b). MorphoBank Project.

[ref-11] Bieler R, Mikkelsen PM, Lee T, Ó Foighil  D (2004). Discovery of the Indo-Pacific oyster *Hyotissa hyotis* (Linnaeus, 1758) in the Florida Keys (Bivalvia: Gryphaeidae). Molluscan Research.

[ref-12] Bieler R, Petit RE (2010). *Thylacodes*–*Thylacodus*–*Tulaxodus*: worm-snail name confusion and the status of *Serpulorbis* (Gastropoda: Vermetidae). Malacologia.

[ref-13] Bieler R, Petit RE (2011). Catalogue of Recent and fossil “worm-snail” taxa of the families Vermetidae, Siliquariidae, and Turritellidae (Mollusca: Caenogastropoda). Zootaxa.

[ref-14] Bohnsack JA, Sutherland DL (1985). Artificial reef research: a review with recommendations for future priorities. Bulletin of Marine Science.

[ref-15] Broughton K (2012). Office of National Marine Sanctuaries science review of artificial reefs. Marine Sanctuaries Conservation Series ONMS-12-05.

[ref-16] Bulleri F, Airoldi L (2005). Artificial marine structures facilitate the spread of a non-indigenous green alga, *Codium fragile* ssp. *tomentosoides*, in the north Adriatic Sea. Journal of Applied Ecology.

[ref-17] Cairns SD (2000). A revision of the shallow-water azooxanthellate Scleractinia of the Western Atlantic. Studies on the Natural History of the Caribbean Region.

[ref-18] Calvo M, Templado J (2005). Reproduction and sex reversal of the solitary vermetid gastropod *Serpulorbis arenarius*. Marine Biology.

[ref-19] Carlton T (1999). Molluscan invasions in marine and estuarine communities. Malacologia.

[ref-20] Carlton JT, Ruckelshaus MH, Simberloff D, Schmitz DC, Brown TC (1997). Nonindigenous marine invertebrates and algae of Florida. Strangers in paradise: impact and management of nonindigenous species in Florida.

[ref-21] Carvalho S, Moura A, Curdia J, Da Fonseca LC, Santos MN (2013). How complementary are epibenthic assemblages in artificial and nearby natural rocky reefs?. Marine Environmental Research.

[ref-22] Chesler J (1994). Not just bilge water. American Conchologist.

[ref-23] Colgan MW, Gabrie C, Harmelin M (1985). Growth rate reduction and modification of a coral colony by a vermetid mollusc, *Dendropoma maxima*.

[ref-24] Colgan DJ, Ponder WF, Beacham E, Macaranas J (2007). Molecular phylogenetics of Caenogastropoda (Gastropoda: Mollusca). Molecular Phylogenetics and Evolution.

[ref-25] Creed JC, Fenner F, Sammarco P, Cairns S, Capel K, Junqueira AOR, Cruz I, Miranda RJ, Carlos-Junior L, Mantelatto MC, Oigman-Pszczol S (2016). The invasion of the azooxanthellate coral *Tubastraea* (Scleractinia: Dendrophylliidae) throughout the world: history, pathways and vectors. Biological Invasions.

[ref-26] Cribb TH, Crespo-Picazo JL, Cutmore SC, Stacy BA, Chapman PA, García-Párraga D (2017). Elucidation of the first definitively identified life cycle for a marine turtle blood fluke (Trematoda: Spirorchiidae) enables informed control. International Journal for Parasitology.

[ref-27] Dodrill J, Fletcher PJ, Kruczynski WL, Fletcher PJ (2012). Florida has an active artificial reef program. Tropical connections: South Florida’s marine environment.

[ref-28] Donahue S, Acosta A, Akins L, Ault J, Bohnsack J, Boyer J, Callahan M, Causey B, Cox C, Delaney J, Delgado G, Edwards K, Garrett G, Keller B, Kellison GT, Leeworthy VR, MacLaughlin L, McClenachan L, Miller MW, Miller SL, Ritchie K, Rohmann S, Santavy D, Pattengill-Semmens C, Sniffen B, Werndli S, Williams DE (2008). The state of coral reef ecosystems of the Florida Keys. In: Wadell JE, Clarke AM, eds. The state of coral reef ecosystems of the United States and Pacific freely associated States. NOAA technical memorandum NOS NCCOS, 73.

[ref-29] EPA (US Environmental protection Agency) (2006). National guidance: best management practices for preparing vessels intended to create artificial reefs (EPA842-B-06-002). https://www.epa.gov/sites/production/files/2015-09/documents/artificialreefguidance.pdf.

[ref-30] Fenner D, Banks K (2004). Orange Cup Coral *Tubastraea coccinea* invades Florida and the Flower Garden Banks, Northwestern Gulf of Mexico. Coral Reefs.

[ref-31] Ferry R (2009). Range expansion of an invasive coral species into South Florida and the Florida Keys National Marine Sanctuary: investigating the ecological impact and source of the invasion.

[ref-32] Garcia EF (1996). The rediscovery of *Cyclothyca corrugata* Stearns, 1890. American Conchologist.

[ref-33] Glasby TM, Connell SD, Holloway MG, Hewitt CK (2007). Nonindigenous biota on artificial structures: could habitat creation facilitate biological invasions?. Marine Biology.

[ref-34] Golding RE, Bieler R, Rawlings TA, Collins TM (2014). Deconstructing *Dendropoma*: a systematic revision of a world-wide worm-snail group with description of new genera (Caenogastropoda: Vermetidae). Malacologia.

[ref-35] Harris LE, Woodring MP (2001). Artificial reefs for submerged and subaerial habitat protection, mitigation and restoration.

[ref-36] Harry HW (1985). Synopsis of the supraspecific classification of living oysters (Bivalvia: Gryphaeidae and Ostreidae). The Veliger.

[ref-37] Harry HW (1986). Oysters of the northwestern Gulf of Mexico. Texas Conchologist.

[ref-38] Healy JM, Mikkelsen PM, Bieler R (2015). Sperm ultrastructure in honeycomb (foam) oysters (Mollusca, Bivalvia, Gryphaeidae, Pycnodontinae): comparison with other Ostreoidea and taxonomic implications. Invertebrate Biology.

[ref-39] Hebert PDN, Cywinska A, Ball SH, DeWaard JR (2003). Biological identifications through DNA barcodes. Proceedings of the Royal Society B-Biological Sciences.

[ref-40] Huber M (2010). Compendium of bivalves. A full-color guide to 3,300 of the world’s marine bivalves. A status on Bivalvia after 250 years of research.

[ref-41] Ingrao DA, Mikkelsen PM, Hicks DW (2001). Another introduced marine mollusk in the Gulf of Mexico: the Indo-Pacific green mussel, *Perna viridis*, in Tampa Bay, Florida. Journal of Shellfish Research.

[ref-42] Jenkins CN, Van Houtan KS (2016). Global and regional priorities for marine biodiversity protection. Biological Conservation.

[ref-43] Johannesson K (1988). The paradox of Rockall: why is a brooding gastropod (Littouina saxatilis) more widespread than one having a planktonic larval dispersal stage (*L. littouea*)?. Mauine Biology.

[ref-44] Johns GM, Kruczynski WL, Fletcher PJ (2012). Artificial reefs have economic value. Tropical connections: South Florida’s marine environment.

[ref-45] Kappner I, Al-Moghrabi SM, Richter C (2000). Mucus-net feeding by the vermetid gastropod *Dendropoma maxima* in coral reefs. Marine Ecology Progress Series.

[ref-46] Katoh S, Standley DM (2013). MAFFT multiple sequence alignment software version 7: improvements in performance and usability. Molecular Biology and Evolution.

[ref-47] Kelly III WC (2007). Three new vermetid gastropod species from Guam. Micronesica.

[ref-48] Klöppel A, Brümmer F, Schwabe D, Morlock G (2013). Detection of bioactive compounds in the mucus nets of *Dendropoma maxima*, Sowerby 1825 (Prosobranch Gastropod Vermetidae, Mollusca). Journal of Marine Biology.

[ref-49] Layton KKS, Martel AL, Hebert PDN (2014). Patterns of DNA barcode variation in Canadian marine molluscs. PLOS ONE.

[ref-50] Lee HG (2011). *Amathina* in the western Atlantic—or—What is *Cyclothyca pacei*?. The Shell-O-Gram.

[ref-51] Leeworthy VR (2011). The economic impact of the USS Vandenberg on the Monroe County Economy. http://sanctuaries.noaa.gov/science/socioeconomic/floridakeys/pdfs/vandenbergreport.pdf.

[ref-52] Leeworthy VR, Maher T, Stone EA (2006). Can artificial reefs alter user pressure on adjacent natural reefs?. Bulletin of Marine Science.

[ref-53] Leray M, Yang JY, Meyer CP, Mills SC, Agudelo N, Ranwez V, Boehm JT, Machida RJ (2013). A new versatile primer set targeting a short fragment of the mitochondrial COI region for metabarcoding metazoan diversity: application for characterizing coral reef fish gut contents. Frontiers in Zoology.

[ref-54] Lindberg WJ, Seaman W (2011). Guidelines and management practices for artificial reef siting, use, construction, and anchoring in Southeast Florida. https://www.dep.state.fl.us/coastal/programs/coral/reports/MICCI/MICCI_18_19.pdf.

[ref-55] Maddison WP, Maddison DR (2017). http://mesquiteproject.org.

[ref-56] Mikkelsen PM, Bieler R, Harper EM, Taylor JD, Crame JA (2000). Marine bivalves of the Florida Keys: discovered biodiversity. Evolutionary biology of the Bivalvia.

[ref-57] Mikkelsen PM, Bieler R (2004). Critical catalog and annotated bibliography of marine bivalve records for the Florida Keys. Malacologia.

[ref-58] Mikkelsen PM, Bieler R (2007). Seashells of Southern Florida–living marine mollusks of the Florida Keys and adjacent regions: Bivalves.

[ref-59] National Ocean Service (2015). Florida Keys National Marine Sanctuary. Shipwreck Trail. http://floridakeys.noaa.gov/shipwrecktrail/.

[ref-60] Odé H (1980). Distribution and records of the marine Mollusca in the northwest Gulf of Mexico. Texas Conchologist.

[ref-61] O’Foighil D (1989). Planktotrophic larval development is associated with a restricted geographic range in *Lasaea*, a genus of brooding, hermaphroditic bivalves. Marine Biology.

[ref-62] Peirano A (2013). Wrecks on the bottom: useful, ecological sentinels?. Marine Technology Society Journal.

[ref-63] Perkol-Finkel S, Shashar N, Benayahu Y (2006). Can artificial reefs mimic natural reef communities? The roles of structural features and age. Marine Environmental Research.

[ref-64] Petuch EJ (1987). New Caribbean molluscan faunas.

[ref-65] Precht WF, Hickerson EL, Schmahl GP, Aronson RB (2014). The invasive coral *Tubastraea coccinea* (Lesson, 1829): implications for natural habitats in the Gulf of Mexico and the Florida Keys. Gulf of Mexico Science.

[ref-66] Rawlings TA, MacInnis MJ, Bieler R, Boore JL, Collins TM (2010). Sessile snails, dynamic genomes: gene rearrangements within the mitochondrial genome of a family of caenogastropod mollusks. BMC Genomics.

[ref-67] Rosenberg G (2009). Malacolog 4.1.1: A Database of Western Atlantic Marine Mollusca. http://www.malacolog.org/.

[ref-68] Ruiz GM, Freestone AL, Fofonoff PW, Simkanin C, Wahl M (2009). Habitat distribution and heterogeneity in marine invasion dynamics: the importance of hard substrate and artificial structure. Marine hard bottom communities.

[ref-69] Sammarco PW, Atchison A, Boland GS (2004). Offshore oil and gas platforms and expansion of coral communities within the Northern Gulf of Mexico. Marine Ecology Progress Series.

[ref-70] Sammarco PW, Lirette A, Tung YF, Boland GS, Genazzio M, Sinclair J (2014). Coral communities on artificial reefs in the Gulf of Mexico: standing vs. toppled oil platforms. ICES Journal of Marine Science.

[ref-71] Scheltema RS (1971). Larval dispersal as a mean of genetic exchange between geographically separated populations of shallow-water benthic marine gastropods. The Biological Bulletin.

[ref-72] Scheltema RS (1972). Eastward and westward dispersal across the Tropical Atlantic Ocean of larvae belonging to the genus *Bursa* (Prosobranchia, Mesogastropoda, Bursidae). International Review of Hydrobiology.

[ref-73] Schofield PJ (2009). Geographic extent and chronology of the invasion of non-native lionfish (*Pterois volitans* [Linnaeus 1758] and *P. miles* [Bennett 1828]) in the Western North Atlantic and Caribbean Sea. Aquatic Invasions.

[ref-74] Seaman W (2004). Artificial reef monitoring in Florida coastal counties. Florida Sea grant college program.

[ref-75] Shearer T (2010). Distribution of *Tubastraea coccinea* in Florida and Flower Garden Banks: progress report.

[ref-76] Sheehy D, Vik SF (2010). The role of constructed reefs in non-indigenous species introductions and range expansions. Ecological Engineering.

[ref-77] Shima JS, Osenberg CW, Stier AC (2010). The vermetid gastropod *Dendropoma maximum* reduces coral growth and survival. Biology Letters.

[ref-78] Shima JS, Phillips NE, Osenberg CW (2013). Consistent deleterious effects of vermetid gastropods on coral performance. Journal of Experimental Marine Biology and Ecology.

[ref-79] Shima JS, Phillips NE, Osenberg CW (2016). Variation in the growth and survival of the tropical vermetid gastropod *Ceraesignum maximum* is driven by size, habitat, and density. Marine Biology.

[ref-80] Simberloff D, Simberloff D,  Schmitz DC, Brown TC (1997). The Biology of invasions. Strangers in paradise: impact and management of nonindigenous species in Florida.

[ref-81] Simone LRL (2011). Phylogeny of the Caenogastropoda (Mollusca), based on comparative morphology. Arquivos de Zoologia.

[ref-82] Simone LRL, Mikkelsen PM, Bieler R (2015). Comparative anatomy of selected marine bivalves from the Florida Keys, with notes on Brazilian congeners (Mollusca: Bivalvia). Malacologia.

[ref-83] Stearns REC (1891). Scientific results of expeditions of the US Fish Commissions Steamer Albatross. Proceedings of the United States National Museum.

[ref-84] Stier AC, McKeon CS, Osenberg CW, Shima JS (2010). Guard crabs alleviate deleterious effects of vermetid snails on a branching coral. Coral Reefs.

[ref-85] Strathmann MF, Strathmann RR (2006). A vermetid gastropod with complex intracapsular cannibalism of nurse eggs and sibling larvae and a high potential for invasion. Pacific Science.

[ref-86] Swofford DL (2002). PAUP*. Phylogenetic analysis using parsimony (*and other methods). Version 4.

[ref-87] Takano T, Kano Y (2014). Molecular phylogenetic investigations of the relationships of the echinoderm-parasite family Eulimidae within Hypsogastropoda (Mollusca). Molecular Phylogenetics and Evolution.

[ref-88] Tyrell MC, Byers JE (2007). Do artificial substrates favor nonindigenous fouling species over native species?. Journal of Experimental Marine Biology and Ecology.

[ref-89] Wilson J, Osenberg CW, St. Mary CM, Watson CA, Lindberg WJ (2001). Artificial reefs, the attraction-production issue, and density dependence in marine ornamental fishes. Aquarium Sciences and Conservation.

[ref-90] Winston JE (2012). Dispersal in marine organisms without a pelagic larval phase. Integrative and Comparative Biology.

[ref-91] WoRMS Editorial Board (2017). World register of marine species. http://www.marinespecies.org/.

[ref-92] Zou SM, Li Q, Kong LF (2011). Additional gene data and increased sampling give new insights into the phylogenetic relationships of Neogastropoda, within the caenogastropod phylogenetic framework. Molecular Phylogenetics and Evolution.

[ref-93] Zvuloni A, Armoza-Zvuloni R, Loya Y (2008). Structural deformation of branching corals associated with the vermetid gastropod *Dendropoma maxima*. Marine Ecology Progress Series.

